# Ultrahigh-speed violet laser diode based free-space optical communication beyond 25 Gbit/s

**DOI:** 10.1038/s41598-018-31431-4

**Published:** 2018-09-03

**Authors:** Wei-Chun Wang, Huai-Yung Wang, Gong-Ru Lin

**Affiliations:** Graduate Institute of Photonics and Optoelectronics, Department of Electrical Engineering, National Taiwan University, Taipei, 10617 Taiwan Republic of China

## Abstract

Violet laser diode (VLD) based ultrahigh-speed free-space optical (FSO) system is demonstrated for point-to-point data transmission. By directly encoding the VLD with 64-quadrature amplitude modulation discrete multi-tone (64-QAM DMT) data stream for optical wireless communication through 0.5–10 m in free space, the point-to-point VLD-based FSO link allows delivering the 64-QAM DMT data at an ultrahigh bit rate of up to 26.4 Gbps. After receiving with a high-speed p-i-n photodiode, such a VLD-FSO link can provide clear constellation plot with error vector magnitude (EVM) of 8.57%, signal-to-noise ratio (SNR) of 21.34 dB and bit error ratio (BER) of 3.17 × 10^−3^ under forward-error-correction criterion. The EVM increases from 8.8% to 9.4% and the SNR decreases from 21.1 to 20.6 dB to slightly degrade the reachable data rate from 25.8 to 24 Gbit/s with transmission distance lengthening from 3 to 10 m.

## Introduction

Nowadays, the demanded data transmission capacity for indoor or harsh free-space environment has rapidly grown up due to the tremendously increased amount of multimedia streaming and data exchange among devices and networks. However, the existing radio frequency (RF) band has been almost congested with data traffic, which triggers alternative communication scenarios to be comprehensively proposed with more flexibility than ever. Accordingly, the visible light source enabled optical wireless communication (OWC), also known as free-space optical (FSO) transmission, visible light communication (VLC) and light-fidelity (Li-Fi)^1^, has been recognized as a promising technology to provide high-speed data communication in free space with unique advantages such as its entire electromagnetic immunity, long propagation distance and high streaming security. At early stage, light-emitting diodes (LEDs) were comprehensively employed to serve as the OWC or FSO transmitters as it can also be easily integrated with the present lighting facility. Nevertheless, the long lifetime of spontaneously emitted photons severely limits its modulation bandwidth to several tens MHz. Although the divergent output beam of LED benefits from wide lighting coverage, it deteriorates the performance of point-to-point FSO link with huge power dissipation under disoriented transmission.

To improve the allowable modulation bandwidth of the OWC or FSO link, laser diodes (LDs) with shorter photon lifetime (~ps), narrower spectral linewidth, longer coherent distance and larger transmission capacity were lately recognized as promising candidate for promoting the VLC data rate. In 2012, Lin *et al*.^2^ preliminarily employed green and red laser pointers to propose a cost-effective visible laser light communication (VLLC) system over 10 m in free space with allowable bit rate of 500 Mbit/s. Subsequently, Watson *et al*.^3^ reported an OWC system at 2.5 Gbit/s based on the directly modulated gallium nitride (GaN) LD at 422 nm. Afterwards, a bidirectional passively optical network (PON) which combined a 20-km single-mode fiber (SMF) channel with a 15-cm-long red-LD VLC channel was designed by Chen *et al*. to achieve 2.5-Gbit/s transmission^4^. In the meantime, the direct non-return-to-zero on-off keying (NRZ-OOK) modulation of a GaN LD at 450 nm for high-speed VLC up to 4 Gbps was also demonstrated^5^. Not until 2015, an OWC system which enabled delivering the OOK data up to 2.6 Gbit/s was proposed by Lee *et al*.^6^, and similar VLLC link with a 410-nm rigid LD further improved the data rate to 5 Gbit/s in the next year^7^. Alternatively, Shen *et al*. designed an integrated waveguide modulator to externally encode the LD at 448 nm at 1 GHz^8^. In 2016, the error-free data rate of the NRZ-OOK stream carried by the blue LD has ultimately approached 5 Gbit/s^9^.

In addition to the use of NRZ-OOK format, the quadrature amplitude modulation orthogonal frequency-division multiplexing (QAM-OFDM) data format was also introduced with enhanced spectral usage efficiency, improved transmission capacity and robust channel tolerance for the VLLC system. The premier demonstration on GaN LD based point-to-point FSO link with 64-QAM OFDM data transmission at 9 Gbit/s over 5 m was demonstrated by Chi *et al*. in 2015^10^. Singh *et al*. employed a 641-nm red laser pointer to realize 4-QAM OFDM data transmission as high as 10 Gbit/s with lengthening distance over 163 m^11^. In 2016, a worldwide new record on high-speed VLLC system via the directly 16-QAM OFDM encoded blue LD at 14 Gbit/s over 7 m was performed by Huang *et al*.^12^. Based on the same format, the 4-Gbit/s blue LD VLLC link in combination with remote phosphor based color converter was preliminarily reported for white lighting^13^. Subsequently, the universal filtered multi-carrier (UFMC) additive 16-QAM OFDM scheme was implemented in the blue LD based VLLC system, which further suppresses the inter-carrier-interference (ICI) between OFDM subcarriers by effectively eliminating the subcarrier side-lobes with programmable spectral filters. Huang *et al*. used such a format to perform a 16-m-long VLLC link with upgraded transmission rate of 18 Gbit/s^14^, which is superior as compared to the performance of 16.4 Gbit/s for the OFDM data with the same cyclic prefix (CP) before adding side-lobe filter^15^.

Later on, the GaN blue LD based point-to-point VLLC has also shown its superiority for underwater wireless optical communication (UWOC), including small divergent angle, long propagation distance and low absorption in seawater environment. Some remarkable UWOC works employed the directly NRZ-OOK modulated green LD at 520 nm with 2.3-Gbit/s data rate^16^, and a 20-m-long UWOC link with 1.5-Gbps NRZ-OOK was successively proposed^17^. With the use of 16-QAM OFDM format, the 450-nm blue LD based UWOC link can speed up to 4.8 Gbit/s^18^. Furthermore, the UWOC distance can lengthen up to 34.5 m with 2.7-Gbps NRZ-OOK data delivered by a green LD^19^. As late as 2017, Wu *et al*. preliminarily demonstrated a UWOC system over a 1.7-m tap-water link at 12.4 Gbit/s^20^, and the modified UWOC link in seawater environment was reported by Huang *et al*. to further upgrade the data rate to 14.8 Gbit/s over the same distance with using the filtered OFDM data format. The allowable transmission capacity only slightly decreases to 10.8 Gbit/s even with lengthening the transmission distance to 10.2 m^21^. Aside from the blue LD based VLLC scheme, Janjua *et al*. further utilized the fiber-based red/green/blue (RGB) LD module to set up a wavelength division multiplexing white-lighting OWC with achieving the bit rate of up to 4 Gbit/s for a single LD^22^. Nearly-ultraviolet LD with RGB phosphors has also been considered for white-lighting VLC at several Gbit/s^23^ Afterwards, Wu *et al*. elevated the RGB-LD mixed white-light transmission data rate up to 8.8 Gbit/s in 2017 with using free-space lens collimation for the transistor outline can (TO-can) packaged LDs^24^. In the same year, the wavelength division multiplexing approach with the RGB LD mixed UWOC link has been updated to 9.5-Gbit/s transmission over 10 m^25^. More recently, Chi *et al*. preliminarily revealed the capability of 405-nm VLD based point-to-point VLLC for carrying 16-QAM OFDM data at 12 Gbit/s^26^.

Even though, the ultimately high transmission capacity of the VLD has yet to be explored until now, and the breakthrough on the VLD based point-to-point FSO link toward 25 Gbit/s per channel relies strictly on data format optimization and device bandwidth upgradation. In this work, the spectrally filtered 64-quadrature amplitude modulation discrete multi-tone (64-QAM DMT) format is implemented as the data stream to directly encode the VLD for FSO transmission, and an ultrafast photodiode in connection with a broadband amplifier is employed as the receiver to provide high cutoff frequency and low noise figure. With distinctly enhanced transmission and receiving performances, the TO-38-can packaged VLD based point-to-point FSO link is performed over a distance from 0.5 to 10 m, and an ultrahigh transmission capacity of 26.4 Gbit/s is successfully demonstrated by enlarging the transmitting bandwidth of the 64-QAM DMT data up to 4.4 GHz. To further elevate the transmission performance, the 64-QAM DMT data format is spectrally filtered to eliminate the ICI caused by the OFDM side-lobes, and the subcarrier power pre-leveling is also performed to enhance the signal-to-noise ratio (SNR) of the data delivered by high-frequency DMT subcarriers. In addition, the bias current, the encoding bandwidth and the pre-leveling slope are respectively optimized to maximize the transmission capacity of the VLD based FSO link with its bit error ratio (BER) below the criterion of forward error correction (FEC).

## Results

### Output characteristics of the violet laser diode

**Figure 1:**
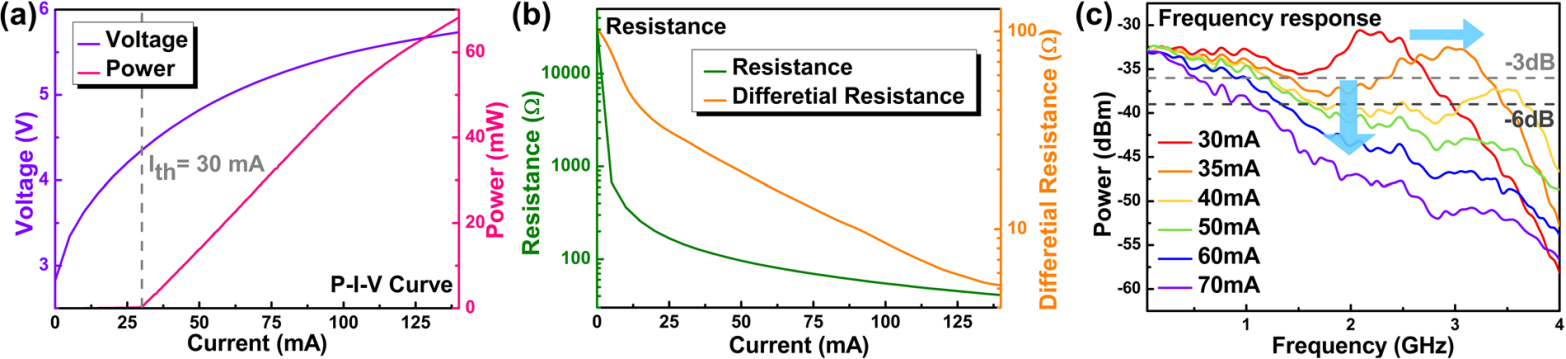
The characteristic of the employed 405-nm VLD. (**a**) The power-current-voltage curve, (**b**) Resistance and differential resistance at different bias currents, and (**c**) frequency response at different bias currents.

With controlling the temperature at 25 °C, the power-current-voltage (P-I-V) curve in Fig. 1(a) for the employed VLD shows a power-to-current slope (*dP/dI*) of 0.6893 W/A beyond its threshold current (*I*_*th*_) of 30 mA, and the available output power can reach 68.2 mW before saturation. The power saturation of VLD starts at biasing beyond 100 mA with 1-dB compressing point at 140 mA. To demand sufficient power for distant point-to-point FSO transmission, the impedance matching must be considered with adjusting the VLD bias current. Both the lumped and differential resistances are calculated by dividing and differentiating the voltage-to-current response, respectively, as shown in Fig. 1(b). The VLD is biased at 60 mA with corresponding differential resistances of 16.3 Ω, which is smaller than 50 Ω typically set for commercially available microwave instruments. Such an impedance mismatch inevitably enlarges the reflection of the data stream directly modulated to the VLD. The reflection coefficient (Γ) of the VLD is calculated as Γ = (Z_load_ − Z_source_)/(Z_load_ + Z_source_) = −0.508. Furthermore, the voltage standing wave ratio (VSWR = (1 + |Γ|)/(1 − |Γ|) of 3.065 is obtained to result in the related return loss (η_RL_ = −20log10(|Γ|)) of 5.883 dB. Direct modulating the VLD biased at 14 dBm and 60 mA leads to a throughput power of only 10.39 dBm with nearly −4 dB attenuation. Figure 1(c) exhibits the directly analog modulated throughput response versus VLD bias, in which the grey and black lines denote the power attenuation level at −3 dB and −6 dB, respectively. As the bias current increases from 30 mA to 40 mA, the relaxation oscillation peak shifts from 2.1 GHz to 3.5 GHz. The −3 dB and −6 dB bandwidths of the VLD biased at 60 mA are 0.97 and 1.33 GHz, respectively. Enlarging the direct current (DC) of the VLD initially broadens and flattens the modulated frequency response, and particularly the high frequency throughput is enlarged with the upshifted relaxation peak. Nevertheless, continuously raising the bias current to 80 mA would attenuate the throughput intensity in the opposite way, which degrades the data transmission performance as the roll-off modulation effect occurs accordingly.

### Point-to-point transmission performance of the VLD based FSO communication link

**Figure 2:**
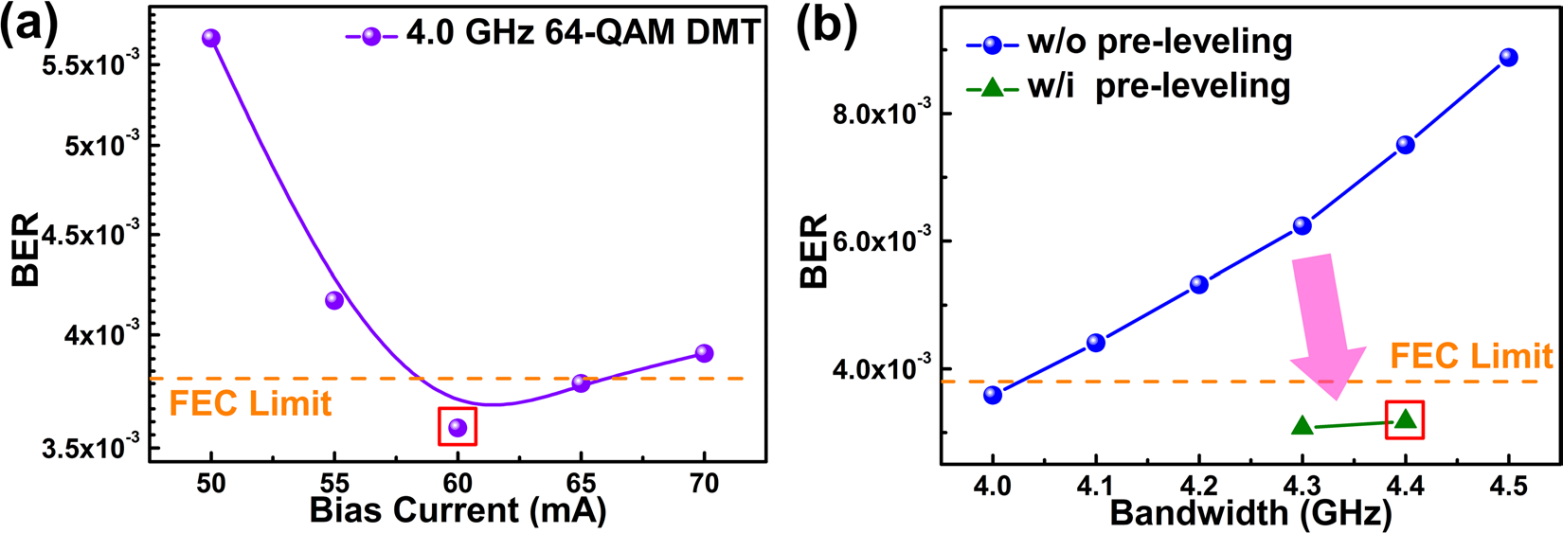
The optimization of the VLD carried 64-QAM DMT data. (**a**) The bias current dependent BERs and (**b**) the bandwidth dependent BERs with and without pre-leveling QAM-DMT data carried by the VLD.

To achieve the maximal data rate for FSO transmission, the 64-QAM DMT data stream was generated from the arbitrary waveform generator (AWG) with a sampling rate of 12 GS/s. After back-to-back and 0.5-m point-to-point FSO transmission, the received data were decoded via an offline MATLAB program. Under broadband QAM-DMT encoding, the VLD bias is optimized to obtain sufficient throughput intensity with suppressed relative intensity noise (RIN). Figure 2(a) indicates the BERs of 4-GHz wide 64-QAM DMT data carried by the VLD with its DC bias adjusted from 50 mA to 70 mA. Increasing the bias current from 50 mA to 60 mA upshifts the relaxation oscillation frequency to improve the signal-to-noise contrast at high frequency region, hence the RIN from the VLD is restrained to improve the BER from 5.67 × 10^−3^ to 3.58 × 10^−3^. Overly biasing the VLD not only declines the modulation throughput but also degrades the on/off extinction ratio, which inevitably increases the BER to 3.91 × 10^−3^ as the VLD bias enlarges to 70 mA. That is, the lowest BER of 3.58 × 10^−3^ can be achieved when biasing the VLD at 60 mA. Subsequently, the bandwidth of 64-QAM DMT data is further expanded to approach the ultimate data rate allowable with the VLD, as the blue sphere symbol in Fig. 2(b) demonstrates. Enlarging the bandwidth from 4 GHz to 4.5 GHz degrades the BER performance from 3.58 × 10^−3^ to 8.89 × 10^−3^, because the high-frequency response of the VLD is insufficient to provide adequate SNR for the demanded FEC criterion. Consequently, the DMT subcarrier power pre-leveling technique is employed to compensate the degraded responses of the high-frequency subcarriers. In detail, the green triangle symbol in Fig. 2(b) represents the received BERs of the pre-leveled 64-QAM OFDM data at 4.3 and 4.4 GHz after delivering by the VLD.

**Figure 3:**
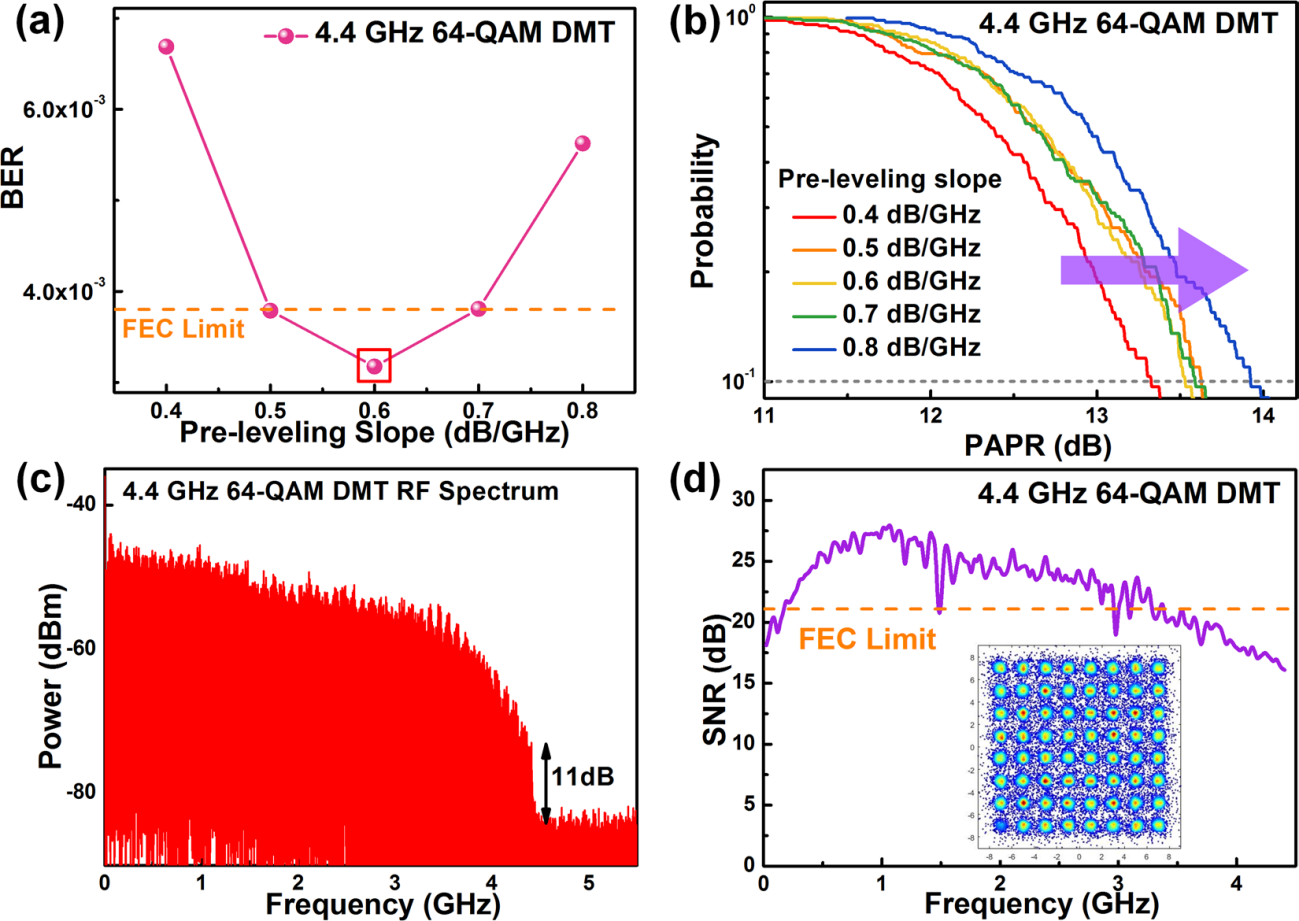
Optimization of the pre-leveling slope and transmission performance of the 4.4-GHz filtered 64-QAM DMT data. (**a**) BERs and (**b**) PAPRs of the filtered 64-QAM DMT data applied with different pre-leveling slopes. (**c**) RF spectrum, (**d**) SNR responses and constellation plots of the filtered 64-QAM DMT data at the bandwidths of 4.4 GHz after pre-leveling.

Later on, to select the appropriate pre-leveling slope for pre-emphasizing the DMT subcarrier amplitude of the 4.4-GHz 64-QAM DMT data stream, the pre-leveling-slope dependent BERs and peak-to-average power ratios (PAPRs) of the received data are measured. Figure 3(a) presents the received BERs enhanced by detuning pre-leveling slope from 0.4 dB/GHz to 0.8 dB/GHz. The pre-leveling slope ought to enlarge for compensating the declined SNRs of data carried by high-frequency subcarriers; however, the SNRs at low-frequency region simultaneously sacrifices by over pre-leveling due to the constant deliverable power from the AWG. Moreover, the probability of PAPR increases when the pre-leveling slope of the pre-emphasized DMT subcarrier amplitude is overly high, which may cause the undesirable nonlinear distortion during post-amplification. As evidence, Fig. 3(b) shows the complementary cumulative distribution functions (CCDFs) of the tested PAPRs. When the pre-leveling slope is enlarged from 0.4 dB/GHz to 0.8 dB/GHz, the PAPR at the probability of 10^−1^ inevitably increases from 13.33 dB to 13.92 dB. Eventually, the lowest BER of 3.17 × 10^−3^ is obtained for the VLD delivered 64-QAM DMT data covering a bandwidth of 4.4 GHz under power pre-leveling at 0.6 dB/GHz. The RF spectrum shown in Fig. 3(c) reveals a carrier-to-noise ratio (CNR) of 11 dB, and the received EVM of 8.57%, SNR of 21.34 dB and BER of 3.17 × 10^−3^ are obtained from the corresponding subcarrier SNRs and the constellation plot shown in Fig. 3(d). To summarize, the VLD bias optimization and QAM-DMT amplitude pre-leveling enable the largest available 64-QAM DMT data bandwidth over 0.5 m in FSO communication link.

### Distant transmission of the VLD based FSO communication link up to 10 m

**Figure 4:**
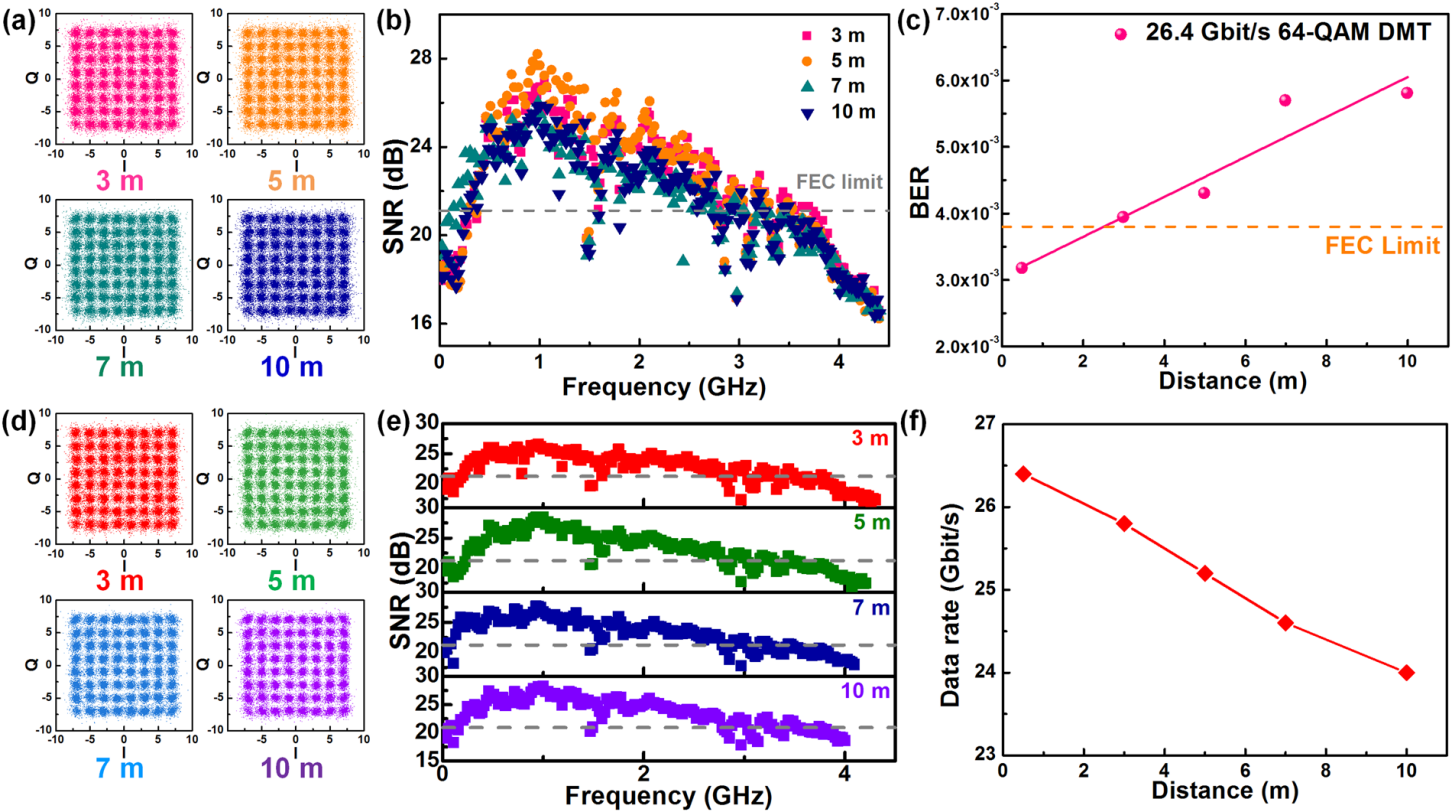
Transmission performance of the VLD carried pre-leveled 64-QAM DMT data over 3 to 10 m. (**a**) The constellation plot, (**b**) the SNR spectra and (**c**) the BERs of 26.4-Gbit/s 64-QAM DMT data transmitting over 3 m, 5 m, 7 m and 10 m. (**d**) The constellation plot, (**e**) the SNR spectra and (**f**) the maximal data rate of 64-QAM DMT data transmitting over 3 m, 5 m, 7 m and 10 m.

At last, the distant dependent transmission with pre-leveled 64-QAM DMT data is performed. First of all, the received constellation plots of the data covering same bandwidth of 4.4 GHz with a total raw data rate of 26.4 Gbit/s over different transmission distances are compared in Fig. 4(a). The estimated EVM for the decoded constellation plot is increased from 8.8% to 9.4% with lengthening the distance from 3 to 10 m. Apparently, the SNR spectra exhibit a degrading tendency which leads to average SNR decreasing from 21.1 to 20.6 dB due to the reduced receiving power with expanding the free-space distance, as shown in Fig. 4(b). The corresponding BER inevitably increases from 3.9 × 10^−3^ to 5.8 × 10^−3^, which cannot pass the FEC criterion after lengthening transmission distance, as shown in Fig. 4(c). That is, the allowable transmission at the longer distant link must be scaled down to the lower bandwidth and data rate under FEC limitation. With shrinking the encoded bandwidth from 4.3 to 4 GHz for lengthening the transmission distance from 3 to 10 m, the received constellation plots and relative SNR spectra are illustrated in Fig. 4(d,e). All the decoded data remain their EVM at 8.8%, average SNR at 21.1 dB and BER below 3.8 × 10^−3^, whereas the maximal data rate reveals a degrading trend from 25.8 to 24 Gbit/s after distance expansion, as shown in Fig. 4(f).

Usually, the turbulent communication channel includes environmental parameters such as raining, snowing, clouding and fogging effects. Under these circumstances, the transmitted light beam would be scattered by vapor, liquid or micro particles to cause receiving power and SNR degradation. Besides, the transmitting path with varying refractive index would be induced to cause beam deflection, chromatic dispersion and data distortion. However, the transmission system with a turbulent communication channel cannot be simulated or performed at present. As the established VLC link in this work cannot play the aforementioned scenarios at current stage, and which also includes a high-speed p-i-n photodiode with very small accepting area. During experiments, the VLD beam must be highly focused onto the photodiode via a double-convex lens to assure the maximal receiving power and SNR. Therefore, such VLD transmission system is less tolerant to mechanic fluctuation and environmental turbulence from this point of view.

## Discussion

With using the directly encoded VLD transmitter, the ultrahigh-speed point-to-point FSO link enables the data rate beyond 25 Gbit/s per single-channel. This is a premier demonstration with directly encoding a TO-can packaged VLD at 405 nm up to 26.4 Gbit/s over 0.5 m in free space for optical wireless communication. By controlling the VLD at room temperature (25 °C) with self-feedback controlled thermo-electric-cooler/thermistor circuit, and matching the impedance of the VLD with adjusting its bias dependent differential resistance, the DC bias current of the VLD is optimized to 60 mA for approaching the FEC qualified BER of the 64-QAM DMT data covering a bandwidth as wide as 4.4 GHz. Both the broadened bandwidth of the receiver and the spectral filtering of the 64-QAM DMT data facilitate such a data capacity enhancement. In particular, the pre-emphasis on the QAM data delivered by high-order DMT subcarriers is executed via a digital power pre-leveling process. With applying the pre-leveling slope of 0.6 dB/GHz, the 4.4-GHz wide 64-QAM DMT data at 26.4 Gbit/s is guaranteed with a FEC-qualified BER of 3.17 × 10^−3^ with an average EVM of 8.57% in constellation plot and an average SNR of 21.34 dB after decoding. By lengthening the distance from 3 to 10 m, the EVM increases from 8.8% to 9.4% and the SNR decreases from 21.1 to 20.6 dB, which inevitably causes the degradation of allowable data rate from 25.8 to 24 Gbit/s with distance expansion. This study experimentally demonstrates the potential application of such a VLD based point-to-point FSO link, which fulfills with need on the compact and cost-effective VLLC system to enable the ultrahigh speed FSO communication in the near future.

## Methods

### The package and temperature control of VLD

**Figure 5:**
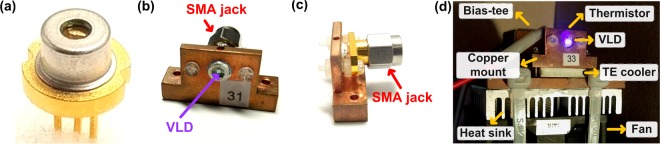
The experimental setup of the 0.5-m point-to-point VLC system.

Figure 5(a) shows the employed TO-can package VLD, which exhibits a threshold current of 30 mA and a lasing wavelength of 405 nm with 3-dB spectral linewidth of 3 nm. It was sealed with a jacket subminiature version A (SMA) connector and mounted with a designed copper heat sink for high-speed modulation and temperature-stabilized operation, as shown in Fig. 5(b,c). Moreover, Fig. 5(d) shows the VLD with self-feedback temperature controlling module. To stabilize the dynamic output of the VLD at controlled temperature, a thermistor is used to sense the instantaneous temperature variation and feedback to the temperature controller (ILX, LDC-3900). Afterwards, the TE cooler installed under the copper mount transfers the heat from VLD to the heat sink so that the heat is balanced between copper mount and heat sink. An electric fan mounted under the heat sink is optionally applied to avoid the overheating via air-cooling. During operation, the self-feedback temperature controlling system, and the temperature maintain at 25 °C with ± 0.05 °C variation.

### Setup of the point-to-point FOC link

**Figure 6:**
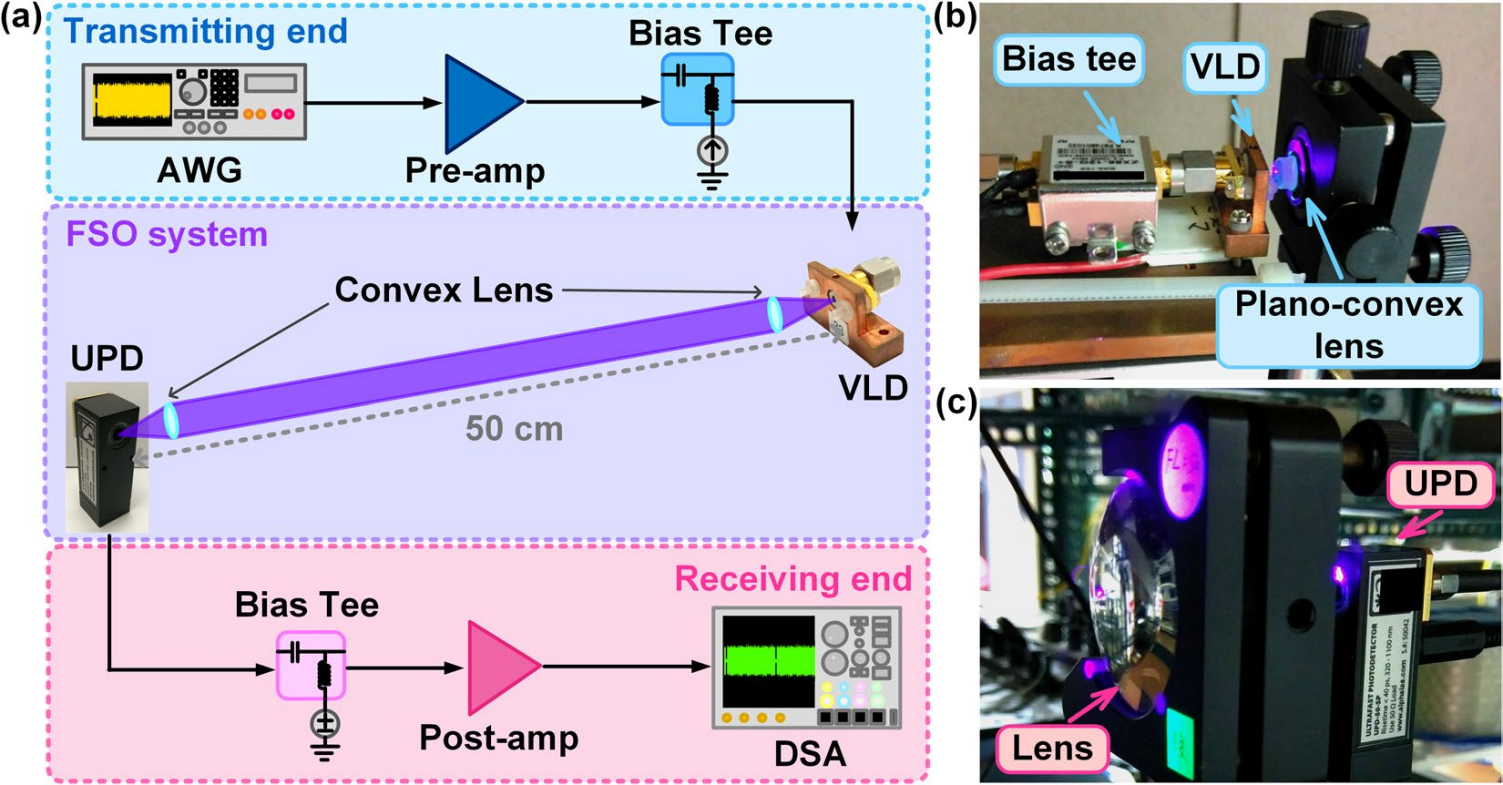
The experimental setup of the 0.5-m point-to-point VLC system.

The setup diagram of the ultrahigh-speed point-to-point FOC link is illustrated in Fig. 6(a). In order to evaluate the allowable transmission capacity, 64-QAM DMT data with a FFT-size of 512 and the programmed band-pass Chebyshev filters set for each subcarrier were employed to avoid the interference between data carried by adjacent subcarriers. During off-line analysis, an AWG (Tektronix, 70001 A) exported the electrical 64-QAM DMT data stream with a peak-to-peak voltage of 500 mV, and a broadband amplifier (Tektronix, 5866) with a gain of 25 dB and a noise figure of 5.75 dB was applied to pre-amplify the data stream to a V_pp_ of 2.2 V. Then, a bias-tee (Mini-circuit, ZX85–12G-S+) combined the data stream with a DC bias current to directly modulate the SMA jacket connected VLD. A plano-convex lens was used to collimate the VLD output from divergent to parallel beam at transmitting end, which exhibits a focal length of 2 mm, a diameter of 2.5 mm and a numerical aperture (NA) of 0.62, as shown in Fig. 6(b). After propagating through 0.5 m in free space, a double-convex lens with a longer focal length of 5 cm, a larger diameter of 5 cm and a smaller NA of 0.5 is employed at receiving end to fully couple the transmitted optical beam into the ultrafast photodiode, as shown in Fig. 6(c). For ultrahigh-speed point-to-point detection, the received signal was detected by an ultrafast photodiode (ALPHALAS, UPD-50 SP) with a conversion bandwidth of 7 GHz. Then, the detected small signal separated from a bias-tee (Picosecond, 55530B) was enhanced by a post-amplifier (Mini-circuit, ZX60-V63+) with a power gain of 20 dB, a bandwidth of 6 GHz and a noise figure of 3.7 dB. Later on, the amplified signal was analyzed by a digital serial analyzer (Tektronix, 71604 C) with a resampling rate of 100 GS/s. Finally, by demodulating the signal with the homemade MATLAB decoding program, the EVM, SNR and BER were estimated.

### Frequency response analyzes

For analog frequency response measurement, a microwave frequency synthesizer (Agilent, E4433B) exported signals with central frequency scanning from 0 GHz to 4 GHz at output power of 0 dBm, and the signal directly modulated on the VLD carrier was transmitting through 0.5 m free-space link. Afterwards, the delivered optical signal was detected by the same ultrafast p-i-n photodiode, and analyzed by a RF spectrum analyzer (Agilent, 8565E) with a bandwidth of 50 GHz. The necessary calibration was done by excluding the impulse response of the testing system and cables.
